# Comparative Study of Pregnancy Outcomes in Women With COVID-19 Disease During the Three Waves of the Pandemic in Eastern India

**DOI:** 10.7759/cureus.67021

**Published:** 2024-08-16

**Authors:** Anisha Choudhary, Archana Barik, Vinita Singh, Abhijeet S Gorwadkar, Mamta R Datta, Alokananda Ray, Mousumi D Ghosh

**Affiliations:** 1 Obstetrics and Gynecology, Tata Main Hospital, Jamshedpur, IND

**Keywords:** third wave, second wave, first wave, preterm, maternal outcome, pregnancy, pandemic, covid-19

## Abstract

Introduction

Healthcare systems around the world were disrupted by the COVID-19 pandemic. Multiple waves were experienced by most countries, and clinical symptoms and severity varied between these waves. A COVID-19 infection in pregnant women may result in complications for both the mother and the fetus and thus pose an additional challenge for clinicians. The study of the different presentations, complications, and pregnancy outcomes during the three waves is important to study the effect of the disease on pregnant women.

Objective

This study aimed to analyze and compare the clinical presentations, comorbid conditions, complications, and pregnancy outcomes in women with SARS-CoV-2 infection during the three waves of the pandemic.

Methodology

The present study is a comparative study undertaken at Tata Main Hospital, a referral hospital in Jamshedpur in eastern India. The study period was from May 2020 to February 2022 and was divided according to the three waves of the pandemic. The duration of the first wave was between 1^st^ May 2020 and 28^th ^February 2021; the second wave was between 1^st^ March 2021 and 31^st ^October 2021; and the third wave was between 1^st ^November 2021 and 28^th ^February 2022. A total of 306 pregnant women tested positive for COVID-19 disease during the study period. A retrospective collection of data was done, and clinical findings, laboratory results, comorbid conditions, and outcomes were compared across the three waves.

Results

During the first wave of the pandemic, 139 COVID-19-positive pregnant women were admitted to our hospital. During the second wave, 110 admitted pregnant women tested positive for SARS-CoV-2 infection, and during the third wave, 57 pregnant women tested positive for SARS-CoV-2 infection. Asymptomatic or mild disease was the most commonly seen presentation during all the waves, but a significantly higher number of moderate and severe cases were seen during the second wave. The second wave also witnessed a higher rate of cesarean sections when compared to the other two waves. The preterm delivery rate was 27.8%, 24.7%, and 25% during the first, second, and third waves of the pandemic, respectively. The third wave of the pandemic had the highest percentage of stillbirths, which was significantly higher than both the first and second waves. The COVID-19 test was positive in four babies during the study period.

Conclusion

The severity of COVID-19 disease varied among the three waves, and the second wave recorded the maximum number of moderate and severe cases. Maternal mortality was also significantly higher during the second wave. The rate of preterm deliveries was high during all the waves, and the incidence of stillbirths was highest during the third wave.

## Introduction

The initial cases of the coronavirus infection occurred in Wuhan, China, in December 2019 and January 2020 [1], and thereafter it spread rapidly across the globe. The World Health Organization (WHO) declared COVID-19 a pandemic on March 11, 2020 [2]. The first case of COVID-19 in India was reported on January 30, 2020, in Kerala [3], after which India entered the first wave of the pandemic. The second wave of the pandemic began in March 2021 and was much more severe and devastating than the first, with a shortage of healthcare facilities and medical supplies throughout the country. The Omicron-dominant third wave began in January 2022 with high infectivity but low severity of the disease [4].

At the beginning of the pandemic, very little was known about the effect of COVID-19 disease on pregnancy. It was expected that pregnant women would suffer from more severe symptoms, as was experienced earlier in pregnant women with severe acute respiratory syndrome (SARS) and Middle East respiratory syndrome (MERS) infections [5,6]. The initial evidence during the first wave reported that pregnant women with SARS-CoV-2 infection did not suffer from more severe disease as compared to non-pregnant women of the same age group [7]. However, as the pandemic continued, findings from a large systemic review and meta-analysis of global data suggested that COVID-19 disease in pregnancy is associated with a higher incidence of severe infection, need for ventilation, and mortality [8]. Several studies have also concluded that the severity of the disease was significantly higher during the second wave as compared to the first wave [9-11]. Mahajan et al. conducted a study in Mumbai, India, and concluded that pregnant women with COVID-19 disease were more severely affected during the first and second waves when compared to the third wave [12].

There is a paucity of research on the effect of COVID-19 disease on pregnancy, especially in eastern India. In this study, we present a comparative analysis of disease presentation, comorbidities, severity, and pregnancy outcomes in women affected by COVID-19 through the three waves of the pandemic.

## Materials and methods

The present study is a retrospective observational study of the pregnancy outcomes of women with SARS-CoV-2 infection admitted to the COVID-19 wing at Tata Main Hospital, a referral hospital in Jamshedpur in eastern India, from May 2020 to February 2022. The study period was divided into three according to the occurrence of the different waves of the pandemic. The duration of the first wave was between 1^st^ May 2020 and 28^th^ February 2021; the second wave was between 1^st^ March 2021 and 31^st^ October 2021; and the third wave was between 1^st^ November 2021 and 28^th^ February 2022.

In accordance with national testing guidelines, all pregnant women visiting the hospital were tested for SARS-CoV-2 infection [13] and categorized into mild, moderate, and severe COVID-19 disease [14]. A total of 306 pregnant women tested positive for SARS-CoV-2 during the study period, and data about their clinical presentation, laboratory findings, management, complications, and pregnancy outcome was retrospectively entered in a Microsoft Excel sheet (Microsoft Corp., Redmond, WA). The data were expressed as frequency (percentage) or median and standard deviation. Comparative analysis of different waves was done by using the Mann-Whitney U test for continuous variables and Pearson’s chi-square test or Fisher's exact test for categorical variables. The confidence interval was 95%, and a p-value <0.05 was considered statistically significant.

## Results

**Figure 1:**
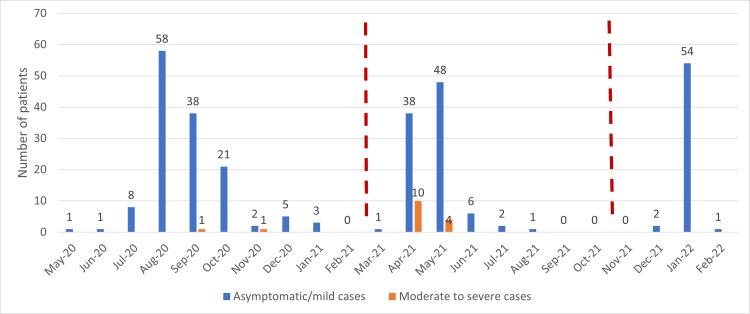
Timelines of the first, second, and third waves of the pandemic The red dashed lines divide the timeline into the three pandemic waves.

During the study period, 306 pregnant women tested positive for COVID-19 infection. The first wave of the pandemic was considered between 1^st^ May 2020 and 28^th^ February 2021, during which 139 COVID-19-positive pregnant women were admitted to our hospital. During the second wave (1^st^ March 2021 to 31^st^ October 2021), 110 admitted pregnant women tested positive for the SARS-CoV-2 infection, and during the third wave (1^st^ November 2021 to 28^th^ February 2022), 57 pregnant women tested positive for the SARS-CoV-2 infection. As shown in Figure 1, the first wave lasted the longest and peaked during the months of August 2020 to October 2020. This was followed by a sudden, sharp rise in the number of cases during the months of April and May 2021, marking the second wave of the pandemic. The number of moderate and severe cases was also significantly higher in the second wave when compared to the first and third waves. The Omicron virus-dominant third wave was the shortest, with no moderate or severe cases.

The mean age of the study group was 27.45±4.51 years, 28.45±4.45 years, and 28.45±4.81 years during the first, second, and third waves, respectively. Pregnant women during the second wave were admitted at a lower period of gestation when compared to those during the first wave (Table 1).

**Table 1:** Demographic profile of pregnant women with COVID-19 infection during the three waves of the pandemic SD: standard deviation ^*^Significant at p-value <0.05

Parameter	First wave	Second wave	Third wave	p-value		
	Mean (SD)	Mean (SD)	Mean (SD)	First wave vs. second wave	First wave vs. third wave	Second wave vs. third wave
Age (years)	27.45 (4.51)	28.45 (4.45)	28.45 (4.81)	0.100	0.07	0.102
Period of gestation (weeks)	35.83 (6.23)	33.70 (8.10)	35.30 (7.38)	0.037^*^	0.945	0.163

Most of the women with COVID-19 infection during pregnancy remained asymptomatic or had mild symptoms during all the waves of the pandemic. The second wave was more severe than the other two waves and also witnessed four cases of maternal mortality (Table 2).

**Table 2:** Disease severity among pregnant women with SARS-CoV-2 infection during the three waves of the pandemic ^*^Significant at p-value <0.05

Severity	First wave n (%)	Second wave n (%)	Third wave n (%)	p-value		
				First wave vs. second wave	First wave vs. third wave	Second wave vs. third wave
Asymptomatic	52 (37.4)	43 (39.1)	47 (82.5)	0.013^*^	<0.0001^*^	<0.0001^*^
Mild	83 (59.7)	53 (48.2)	10 (17.5)			
Moderate	2 (1.4)	10 (9.1)	0 (0.0)			
Severe	2 (1.4)	4 (3.6)	0 (0.0)			
Mortality	0 (0.0)	4 (3.6)	0 (0.0)	0.037^*^	>0.9999	0.030^*^

Hypertensive disorders (preeclampsia, gestational hypertension, and chronic hypertension) and diabetes (gestational diabetes and type 2 diabetes mellitus) were the most frequently associated comorbidities among pregnant women. Deranged liver enzymes were seen in more than 50% of study subjects during the second wave, which was significantly more than that observed during the first and third waves (Table 3).

**Table 3:** Comorbidities seen among women with COVID-19 infection during pregnancy ^*^Significant at p-value <0.05 DM: diabetes mellitus; GDM: gestational diabetes mellitus; HTN: hypertension; GHTN: gestational hypertension

Comorbidities	First wave n (%)	Second wave n (%)	Third wave n (%)	p-value		
				First wave vs. second wave	First wave vs. third wave	Second wave vs. third wave
DM/GDM	17 (12.23)	16 (14.55)	7 (1.23)	0.593	0.770	0.790
Anemia	12 (8.63)	11 (10)	3 (5.3)	0.711	0.560	0.339
Cardiovascular disease	0 (0.00)	2 (1.82)	0 (0.00)	0.194	>0.99	0.19
HTN/GHTN	18 (12.94)	16 (14.55)	9 (15.8)	0.612	0.87	0.70
Deranged liver enzymes	54 (38.85)	57 (51.82)	21 (36.84)	<0.0001^*^	0.099	<0.0001^*^
Morbid obesity	2 (1.44)	2 (1.82)	1 (1.75)	0.816	0.723	0.812

As seen in Table 4, the cesarean section rate was significantly higher during the second wave (78.8%) when compared to the first (64.6%) and the third wave (52.1%). Two patients during the second wave and one each during the first and third waves had laparotomies for ruptured ectopic pregnancies. The preterm delivery rate was 27.8%, 24.7%, and 25% during the first, second, and third waves of the pandemic, respectively, and this difference did not reach statistical significance. The third wave of the pandemic had the highest percentage of stillbirths (8.3%), which was significantly higher than both the first and second waves. Four babies tested positive for COVID-19 infection after birth, two of them during the second wave and one each during the other two waves.

**Table 4:** Comparative analysis of mode of delivery and neonatal outcomes of COVID-19 disease in pregnant women during the first, second, and third waves of COVID-19 ^*^Significant at p-value <0.05

Parameter	First wave n (%)	Second wave n (%)	Third wave n (%)	p-value		
				First wave vs. second wave	First wave vs. third wave	Second wave vs. third wave
Mode of delivery						
Cesarean section	82 (64.6)	67 (78.8)	25 (52.1)	0.032^*^	0.138	0.014^*^
Vaginal delivery	45(35.4)	18 (21.2)	23 (47.9)			
Ectopic pregnancy	1 (0.7)	2 (1.8)	1 (1.8)	0.12	0.12	0.88
Neonatal outcome						
Preterm	35 (27.8)	21 (24.7)	11 (25.0)	0.620	0.730	0.72
Low birth weight	36 (29.3)	26 (31.0)	14 (31.8)	0.795	0.810	0.910
Still birth	4 (2.9)	2 (1.9)	4 (8.3)	0.699	0.06	0.03^*^
Neonatal death	3 (2.4)	1 (1.2)	0 (0.0)	0.648	0.058	0.781
COVID-19-positive	2 (1.6)	1 (1.2)	1 (2.3)	0.794	0.541	0.991

## Discussion

The COVID-19 pandemic presented multiple challenges to the healthcare system globally. A three-wave pattern of coronavirus infection was seen in most countries during the pandemic. Differences in the severity of the disease have been reported during the three waves; however, data on comparative analysis of the pregnancy outcomes in women with SARS-CoV-2 infection during the three waves are scarce.

The peak of the first wave was seen during the second half of 2020, and the number of cases started to decrease thereafter. The second wave of COVID-19 began in late March 2021 and was more virulent and severe than the first [15]. In November 2021, another heavily mutated B1.1.1.529 variant, also called Omicron, was reported as concerning and responsible for the third wave of the pandemic in our country [16]. In our study, 139 pregnant women tested positive for SARS-CoV-2 infection during the first wave (May 2020 to February 2021). During the second wave (March 2021 to October 2021), 110 admitted pregnant women tested positive for SARS-CoV-2 infection, and during the third wave (November 2021 to February 2022), 57 pregnant women tested positive for SARS-CoV-2 infection. The number of admissions was higher during the first wave when compared to the second and third, but most of these women during the first wave (97%) were asymptomatic or had mild symptoms. The second wave of the COVID-19 pandemic was the most severe among the three waves and witnessed four maternal mortalities. Ambedkar et al. did a comparative analysis of pregnancy outcomes among women with COVID-19 disease during the first and second waves of the pandemic. They reported a higher number of admissions among pregnant women in the first wave when compared to the second wave [17]. Mahajan et al. conducted a study in Mumbai and reported decreased severity, admission to the intensive care unit, case fatality rate, and maternal mortality ratio during the third wave of the pandemic [12]. A higher incidence of severe illness among pregnant women was also recorded in the United Kingdom during the second wave [18].

In our study, the mean age of the study subjects did not statistically differ during the three waves. The most frequently associated comorbidities seen in our study were hypertensive disorders and diabetes. Singh et al. conducted a study in Jaipur, India, and reported that the pregnant women admitted during the second wave were younger, and those admitted during the third wave had significantly more comorbid conditions [15]. Approximately half of the pregnant women with COVID-19 infection had altered liver enzymes during the second wave of the pandemic, which was significantly more than that observed during the first and third waves. Several studies have reported an association between COVID-19 infection and acute liver dysfunction. Drug interactions, septic shock, acute respiratory failure, and multiorgan dysfunction have been described as the causative factors for liver injury in COVID-19-positive patients [19,20]. The extent of liver dysfunction has also been significantly associated with elevated inflammatory markers, prolonged hospital length of stay, and a more severe clinical course [19,21].

In our study, a significantly higher percentage of pregnant women had a cesarean section when compared to the other two waves. Various studies across the globe have reported a high cesarean section rate among pregnant women infected with SARS-CoV-2 [10,18]. Silva et al. reported that this increase is mainly due to maternal requests and fear concerning the outcome of the disease [22]. The preterm birth rate remained high during all three waves (27.8%, 24.7%, and 25% during the three waves, respectively). Many studies have reported that COVID-19 infection in pregnant women raises the risk of preterm birth, but the data are still conflicted on whether these are iatrogenic or spontaneous preterm deliveries [10,23,24]

Neonatal COVID-19 infection was documented in two babies during the first wave, one during the second wave, and one during the third wave, respectively. All four babies did not develop any symptoms and were roomed in along with their mothers. Chaudhary et al. reported neonatal COVID-19 infection in five babies during their study period [10]. There were few initial reports against the possibility of vertical transmission of the SARS-CoV-2 infection [25]. However, recent literature shows that vertical transmission of SARS-CoV-2 is possible and seems to occur in a minority of cases [26].

The limitations of the study include that it is a single-center study with a comparatively small sample size. We could not assess the different SARS-CoV-2 variants during the three waves of the pandemic due to the non-availability of genome sequencing. We also lack follow-up to assess the long-term sequelae of the disease on the mother and the neonate.

## Conclusions

Our study concludes that the COVID-19 infection in pregnancy may present with varied symptoms, complications, and severity. The first wave lasted longer than the other two waves; however, the majority of patients remained asymptomatic during the first wave. The second wave was the most severe, and the third wave was the mildest. This variation may be because of the nationwide vaccination drive or the new mutated strain of the virus. All three waves witnessed a high rate of preterm deliveries, and neonatal COVID-19 infection was documented in four babies during the study period, concluding that vertical transmission of COVID-19 infection is a possibility. Further research should be directed toward understanding the course and outcome of the disease among pregnant women. This will help us overcome the unpredictable nature of mutations, and the healthcare sector will be better equipped to handle such types of infections in the future.
